# The localization of pre mRNA splicing factor PRPF38B is a novel prognostic biomarker that may predict survival benefit of trastuzumab in patients with breast cancer overexpressing HER2

**DOI:** 10.18632/oncotarget.22496

**Published:** 2017-11-18

**Authors:** Tarek M.A Abdel-Fatah, Robert C Rees, A. Graham Pockley, Paul Moseley, Graham R Ball, Stephen Y.T Chan, Ian O Ellis, Amanda K Miles

**Affiliations:** ^1^ Department of Clinical Oncology, Nottingham City Hospital, Nottingham University Hospitals NHS Trust, Nottingham, NG5 1PB, UK; ^2^ The John van Geest Cancer Research Centre, Nottingham Trent University, Nottingham, NG11 8NS, UK; ^3^ Department of Histopathology, Nottingham City Hospital, Nottingham University Hospitals NHS Trust, Nottingham, NG5 1PB, UK

**Keywords:** cancer antigen, PRPF38B, poor prognosis, ER-negative/HER2-positive breast cancer, trastuzumab therapy

## Abstract

Cancer biomarkers that can define disease status and provide a prognostic insight are essential for the effective management of patients with breast cancer (BC).

The prevalence, clinicopathological and prognostic significance of PRPF38B expression in a consecutive series of 1650 patients with primary invasive breast carcinoma were examined using immunohistochemistry. Furthermore, the relationship(s) between clinical outcome and PRPF38B expression was explored in 627 patients with ER-negative (oestrogen receptor) disease, and 322 patients with HER2-overexpressing disease.

Membranous expression of PRPF38B was observed in 148/1388 (10.7%) cases and was significantly associated with aggressive clinicopathological features, including high grade, high mitotic index, pleomorphism, invasive ductal carcinoma of no specific type (IDC-NST), ER-negative, HER2-overexpression and p53 mutational status (all *p* < 0.01). In patients with ER-negative disease receiving chemotherapy, nuclear expression of PRPF38B was significantly associated with a reduced risk of relapse (*p* = 0.0004), whereas membranous PRPF38B expression was significantly associated with increased risk of relapse (*p* = 0.004; respectively) at a 5 year follow-up. When patients were stratified according to ER-negative/HER2-positive status, membranous PRPF38B expression was associated with a higher risk of relapse in those patients that did not receive trastuzumab therapy (*p* = 0.02), whereas in those patients with ER-negative/HER2-positive disease that received trastuzumab adjuvant therapy, membranous PRPF38B expression associated with a lower risk of relapse (*p* = 0.00018).

Nuclear expression of PRPF38B is a good prognostic indicator in both ER-negative patients and ER-negative/HER2-positive BC (breast cancer) patients, whereas membranous localisation of PRPF38B is a poor prognostic biomarker that predicts survival benefit from trastuzumab therapy in patients with ER-negative/HER2-overexpressing BC.

## INTRODUCTION

The incidence of breast cancer in the UK has grown by 50% in the past 25 years, making it the UK's most common form of cancer. In 2010, 49,564 women were diagnosed with breast cancer and 11,684 women died from the disease in 2011. Although the number of diagnosed cases has increased, so has the 5 year survival rate for patients, with almost 85.1% of women surviving 5 years following first diagnosis (www.cancerresearchuk.org). However, despite such increases in the survival rates of women with breast cancer, recurrence occurs in over 20% of patients, as a consequence of which there is an unmet clinical need to identify biomarkers that can stratify patients at the risk of recurrence and identify those patients who optimally respond to therapy [1].

The promotion of cell growth and proliferation contributes to the development and progression of breast cancer. Growth signalling pathways are triggered by a number of membrane bound and intracellular receptors, including the oestrogen receptor, progesterone receptor (PR) and the epidermal growth factor receptor HER2. The expression and biological activities of membrane-bound receptors therefore play a significant role in the initiation and progression of breast cancer, and the prevention, treatment and relapse of the disease [2]. HER2 belongs to the family of epidermal growth factor receptors, the amplification or overexpression of which occurs in 20–25% of human breast cancers [3]. The amplification of HER2 strongly correlates with carcinogenesis and is an independent predictor for the poor prognosis of patients with breast cancer [4–6]. HER2 overexpression is also found at metastatic sites, thereby suggesting that therapies targeting HER2 may be effective for both localized and metastatic disease [7]. This is significant given that approximately 90% of cancer-related deaths are due to aggressive, metastatic disease. To date, four therapies have been approved for the targeting of HER2-positive breast cancer: two antibodies (trastuzumab and pertuzumab), an antibody-drug conjugate (ado-trastuzumab emtansine), and a small molecule kinase inhibitor (lapatinib) [8].

Trastuzumab (Herceptin^®^) was the first candidate drug which was registered for use in patients with breast cancer overexpressing HER2. Although trastuzumab prolongs the survival of HER2-positive breast cancer patients and is the standard of care for HER2-positive breast cancer (NCCN Clinical Practice Guidelines in Oncology Breast Cancer (2012) V. 2), resistance to the drug is common, with the overall response rates (ORRs) with single-agent trastuzumab being in the range of 15–26% [9, 10]. Substantially higher response rates (~50%) have been achieved when trastuzumab is used in combination with standard chemotherapy [11]. Trastuzumab is associated with significant drug acquisition costs [12] and side-effects that include infusion reactions, as well as cardiac and pulmonary toxicity [7]. Given the resistance to therapy, costs and side-effects, the ability to stratify patients on the basis of their clinical responsiveness to trastuzumab therapy would have a significant beneficial impact on the clinical management of patients with HER2-positive breast cancer. With this in mind, the development of an improved understanding of the fundamental mechanisms of trastuzumab action and molecular determinants of response, and the identification of therapeutic agents that can either potentiate the effect of trastuzumab or target cells which have become resistant to trastuzumab have become a major focus for the treatment of HER2-positive breast cancer.

As part of an ongoing programme to identify novel cancer testis antigens that may be associated with cancer, we employed the SEREX (serological analysis of recombinant cDNA expression libraries) methodology to screen cancer patient sera against a normal testicular cDNA library (unpublished results). This screen identified a promising candidate, PRPF38B [GenBank: NP_060531], which belongs to a family of genes that are related to the pre-mRNA splicing factor from yeast and is commonly perceived as being located within the nucleus of cells. [13].

The current study investigated the clinicopathological significance of the expression of PRPF38B in a consecutive series of normal breast tissue (*n* = 40), primary invasive breast carcinomas (*n* = 1650) and ER-negative disease (*n* = 627). Furthermore, the relationship between the expression of PRPF38B and clinical outcome of patients with HER2-positive disease who did not (*n* = 221) or did (*n* = 101) receive trastuzumab has been explored in consecutive cases of a HER2-positive cohort (total *n* = 322) in order to examine a potential association between PRPF38B protein expression and aggressive disease/therapeutic response following trastuzumab treatment.

Our findings suggest that determining the localization of PRPF38B expression in patients with ER-negative and ER-negative/HER2-positive breast cancer has the potential to stratify these patients for therapy. To the best of our knowledge, this is the first study to report on the prognostic and potential predictive value of PRPF38B expression in primary breast cancer.

## RESULTS

### Tissue expression of PRPF38B and its association with clinicopathological features and relevant breast cancer biomarkers

**Figure 1:**
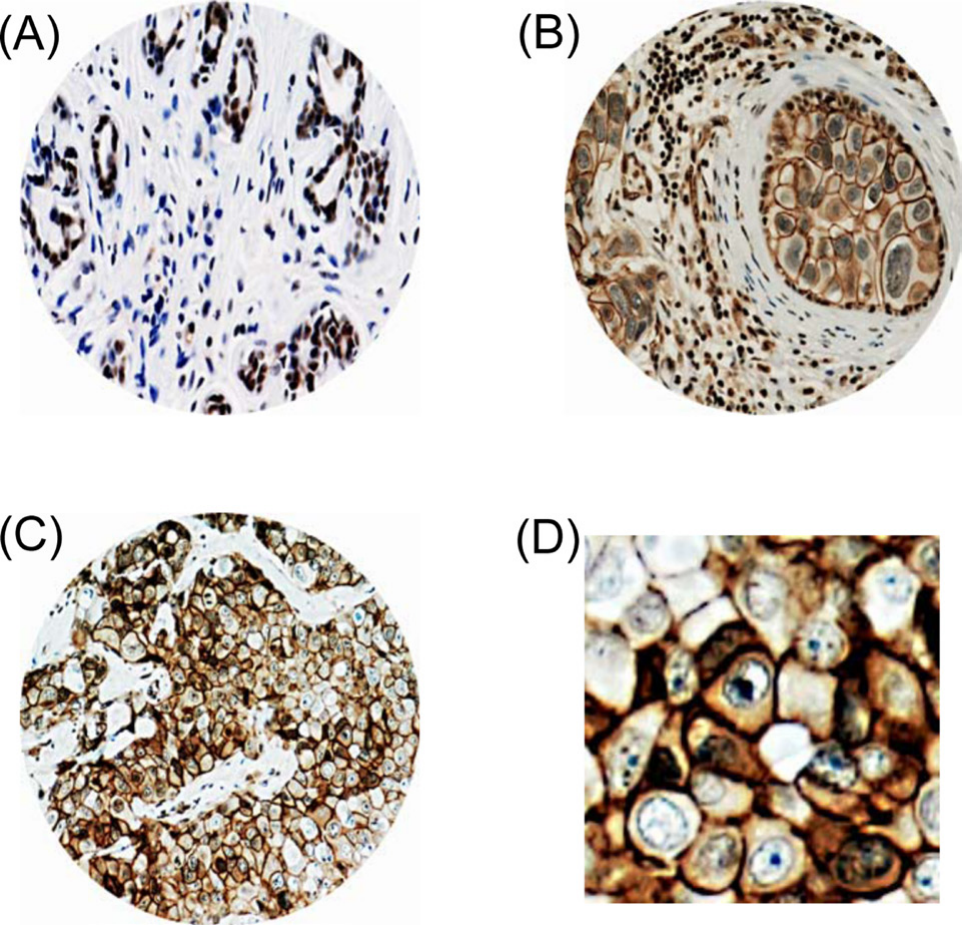
PRPF38B protein expression in breast tissues Representative photomicrographs of PRPF38B expression, as assessed using IHC. (**A**) PRPF38B demonstrated homogenously intense nuclear staining in normal breast tissue. (**B**) PRPF38B expression was localized in the nucleus of peripheral cells and tumour epithelial cells exhibited membranous staining in ductal carcinoma *in situ*. (**C**) High grade invasive breast cancer demonstrated staining of PRPF38B in the cytoplasm and nucleus of cells, with intense membranous staining also being observed [magnification 200× for A–C]. (**D**) Enlarged image of (C) demonstrating intense membranous staining.

In all of the normal tissues examined, PRPF38B was predominately located within the nucleus of cells with some weak cytoplasmic, but no membranous staining (Figure 1A). Out of 1650 cases of early stage invasive breast cancer, 1388 were suitable for the scoring of PRPF38B expression. Eight hundred and ninety tissues (64%) showed no staining for PRPF38B, whereas 148 tissues (10.7%) showed membranous staining and 360 (25.9%) displayed nuclear staining (Figure 1B–1D). Simultaneous expression of both nuclear and membranous was rarely observed (10/1388; 0.7%).

The analysis of 1388 tissue cores that were randomised into 2 approximately equal populations (training and test sets) revealed no significant differences between the 2 populations regarding expression of PRPF38B and other clinicopathological features (Supplementary Table 1).

PRPF38B nuclear staining was statistically associated with the expression of oestrogen and progesterone receptors, but with a lack of HER2 expression. In contrast, membranous PRPF38B staining was associated with aggressive phenotypes including ER-negative, PR-negative, HER-overexpression, high pleomorphism and high tumour grade. Furthermore, membranous PRPF38B expression was significantly associated with the expression of markers that are linked with high proliferation including high mitotic index, high Ki67, p53 mutation and low Bcl2. The summary of these staining patterns relative to these and other clinicopathological correlates is provided in Table 1.

**Table 1:** Clinicopathological characteristics of non-membranous (N-MB) or membranous (MB) PRPF38B

Variables	Training Set (*n* = 688)*n* (%)		*p* value	Test Set (*n* = 688) *n* (%)		*p* value
	N-MB (*n* = 613)	MB (*n* = 75)		N-MB (*n* = 616)	MB (*n* = 72)	
**Pathological parameters**						
**Tumour size**						
T1 a + b (≤1.0)	60 (9.9)	4 (5.5)	0.465	76 (12.8)	5 (7.0)	0.499
T1 c (>1.0–2.0)	319 (52.7)	37 (50.7)		296 (48.1)	34 (47.9)	
T2 (>2.0–5.0)	213 (35.2)	31 (42.5)		223 (36.3)	30 (42.3)	
T3 (>5)	13 (2.1)	1 (1.4)		17 (2.8)	2 (2.8)	
**Lymph node stage**						
Negative	382 (62.8)	38 (51.4)	0.159	367 (59.7)	43 (60.6)	0.010
Positive (1–3 nodes)	175 (28.8)	28 (37.8)		197 (32.0)	15 (21.1)	
Positive (>3 nodes)	51 (8.4)	8 (10.8)		51 (8.3)	13 (18.3)	
**Tumour grade**						
G1	110 (18.2)	4 (5.5)	<0.0001^*^	115 (18.7)	2 (2.8)	<0.0001^*^
G2	218 (36.0)	13 (17.8)		213 (34.6)	16 (22.5)	
G3	277 (45.8)	56 (76.7)		287 (46.7)	53 (74.6)	
**Mitotic index**						
M1 (low; mitoses <10)	246 (40.9)	6 (8.2)	<0.0001^*^	229 (37.4)	10 (14.1)	<0.0001^*^
M2 (medium; mitoses 10–18)	114 (18.9)	16 (21.9)		112 (18.3)	11 (15.5)	
M3 (high; mitoses >18)	242 (40.2)	51 (69.9)		271 (44.3)	50 (70.4)	
**Pleomorphism**						
P1	12 (2.0)	2 (2.7)	0.00^1*^	20 (3.3)	0 (0)	<0.0001^*^
P2	257 (42.7)	14 (19.2)		251 (41.1)	13 (18.3)	
P3	333 (55.3)	57 (78.1)		340 (55.6)	58 (81.7)	
**Tubule formation**						
T1	31 (5.1)	0 (0)	0.003^*^	47 (7.7)	2 (2.8)	0.006^*^
T2	210 (34.9)	15 (20.5)		209 (34.2)	14 (19.7)	
T3	361 (60)	58 (79.5)		356 (58.2)	55 (77.5)	
**Tumour type**						
IDC-NST	301 (55.9)	57 (93.4)	<0.0001^*^	297 (55.8)	53 (84.1)	<0.0001^*^
Medullary/atypical	14 (2.6)	1 (1.6)		11 (2.1)	3 (4.8)	
Tubular carcinoma	121 (22.4)	0 (0)		122 (22.9)	6 (9.5)	
Invasive lobular carcinoma	61 (11.3)	3 (4.9)		54 (10.2)	1 (1.6)	
Others	41 (7.6)			48 (9.0)	0 (0)	
**Hormonal receptors**						
Oestrogen receptor						
Negative	131 (21.8)	47 (65.3)	<0.0001^*^	141 (23.3)	39 (56.5)	<0.0001^*^
Positive	471 (78.2)	25 (34.7)		465 (76.7)	30 (43.5)	
**Progesterone receptor**						
Negative	217 (38.3)	54 (76.1)	<0.0001^*^	218 (37.8)	43 (66.2)	<0.0001^*^
Positive	349 (61.7)	17 (23.9)		359 (62.2)	22 (33.8)	
**HER2**						
Negative	555 (92.2)	32 (43.2)	<0.0001^*^	569 (94.2)	36 (50.7)	<0.0001^*^
Overexpression	47 (7.8)	42 (56.8)		35 (5.8)	35 (49.3)	
**Cell cycle/apoptosis**						
**P53**						
Negative	406 (82.4)	42 (67.7)	0.006^*^	411 (82.0)	30 (48.4)	<0.0001^*^
Positive	87 (17.6)	20 (32.3)		90 (18.0)	32 (51.6)	
**Bcl2**						
Negative	179 (32.5)	41 (59.4)	<0.0001^*^	169 (30.6)	38 (56.7)	<0.0001^*^
Positive	371 (67.5)	28 (40.6)		384 (69.4)	29 (43.3)	

Significant differential distributions between non-membranous (N-MB) and membranous (MB) PRPF38B expression. Membranous PPF38B expression was more prevalent in Grade 3 tumours, invasive ductal carcinoma–no specific type IDC-NST, patients with ER-negative, PR-negative, HER2-positive and Bcl2-negative breast cancers. The frequency of membranous expression was associated with a high mitotic index, a marked variation in pleomorphism and high tubule formation. No significant differential distribution between membranous and non-membranous PRPF38B expression was observed for tumour size and lymph node stage. ^*^Statistically significant differences between the frequency of distribution were calculated using Pearson's Chi-squared analysis with a *p* value < 0.05 considered statistically significant.

### Relationship between membranous PRPF38B expression and clinical outcome of early stage primary invasive breast cancer

**Figure 2:**
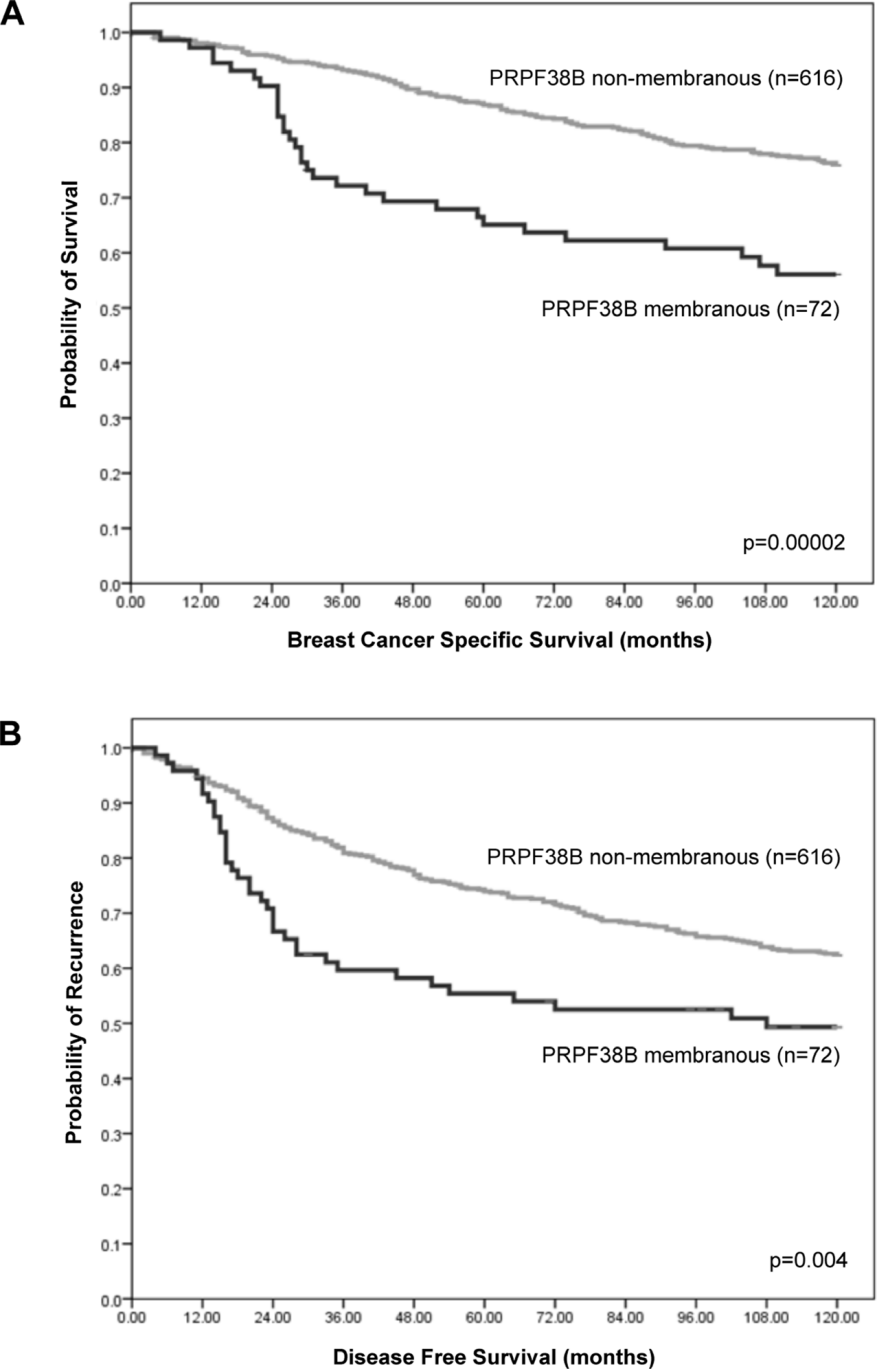
Relationship between cellular PRPF38B expression and survival of patients with breast cancer Membranous PRPF38B expression was significantly associated with poor breast cancer specific survival (BCSS) (log rank = 18.06, *p* = 0.00002) (**A**) and disease-free survival (DFS) in patients with breast cancer (log rank = 8.31, *p* = 0.004) (**B**), as calculated using Kaplan-Meier univariate survival analysis.

Whereas positive nuclear staining was associated with disease-free survival (DFS, data not shown), high membranous expression of PRPF38B was significantly associated with increased risk of relapse (*p* = 0.004) at 10 years (Figure 2).

Multivariate Cox regression analysis including validated prognostic factors (lymph node metastases, histological grade, tumour size, ER receptor positivity and HER2 status) and controlling for both endocrine and chemotherapies, demonstrated that membranous PRPF38B expression was an independent predictor of DFS (training set; HR (hazard ratio) [95% CI (confidence interval)): 1.76 (1.04–2.98, *p* = 0.037 and test set: [HR (95% CI): 1.69 (1.00–2.85, *p* = 0.048)]. Pooling both the training data set and test set together, membranous PRPF38B expression was associated with a 65% increase in the risk of relapse [HR (95% CI): 1.65 (1.14–2.38, *p* = 0.0007)] (Table 2).

**Table 2:** Multivariate Cox regression analysis showing 5 year disease-free survival (DFS) in the training set, test set and all patients set

Variables	Training Set			Test Set			All Patients		
	HR	CI 95%	*p*	HR	CI 95%	*p*	HR	CI 95%	*p*
PRPF38B membranous (+)	1.76	1.04–2.98	0.037^*^	1.69	1.00–2.85	0.048^*^	1.65	1.14–2.38	0.0007^*^
PRPF38B membranous (−)	0.82	0.53–1.27	0.38	0.86	0.55–1.35	0.519	0.85	0.62–1.16	0.294
Lymph node metastases (+)	2.22	1.55–3.16	<0.0001^*^	1.76	1.22–2.54	<0.0001^*^	1.99	1.54–2.57	<0.0001^*^
Histological grade (G1/2 vs 3)	1.47	0.96–2.23	0.074	1.26	1.64–4.00	<0.0001^*^	1.91	1.41–2.58	<0.0001^*^
Size (continuous)	1.23	1.00–1.50	0.05^*^	1.42	1.19–1.70	<0.0001^*^	1.35	1.18–1.54	<0.0001^*^
ER (+)	0.67	0.45–1.02	0.059	0.90	0.59–1.38	0.624	0.77	0.57–1.03	0.078
HER2 (+)	1.22	0.75–1.97	0.42	1.05	0.66–1.66	0.837	0.6	0.81–1.57	0.463
Chemotherapy	0.53	0.30–0.93	0.026^*^	.077	0.49–1.22	0.27	0.58	0.40–85	0.005^*^
Endocrine therapy	0.47	0.29–76	0.002^*^	0.60	0.35–1.03	0.065	0.62	0.45–86	0.004^*^

^*^Statistical significance was calculated using multivariate Cox regression analysis (*p* < 0.05 was considered significant). Other validated traditional prognostic factors and cofounders were included in the model, and the model was controlled for endocrine and chemotherapy. HR; hazard ratio, CI; confidence interval, ER; oestrogen receptor.

### Relationship between cellular localization of PRPF38B expression and clinical outcome for patients with ER-negative breast cancer

**Figure 3:**
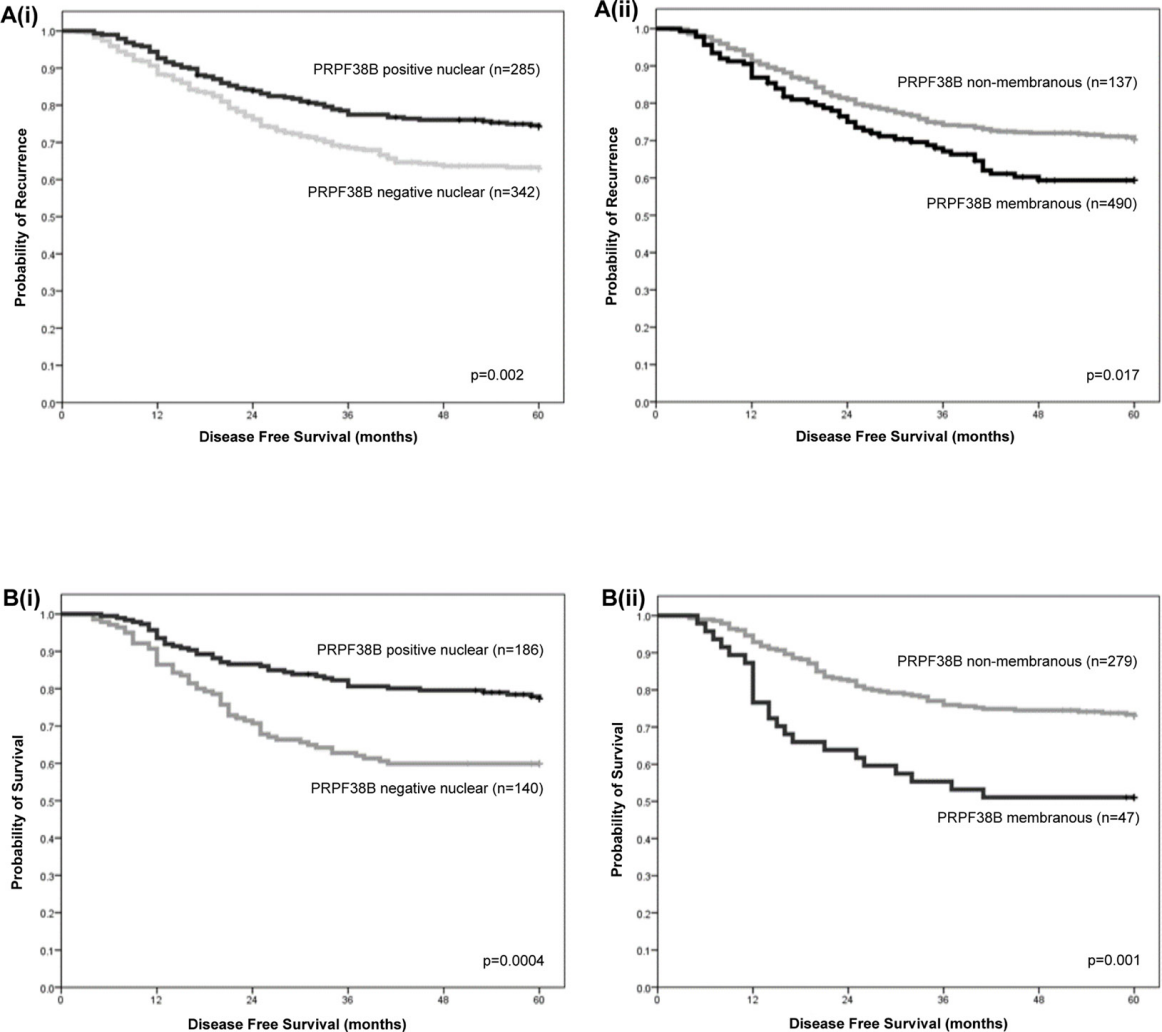
Relationships between nuclear and membranous PRPF38B expression and clinical outcome in patients with ER-negative breast cancer and patients with ER-negative breast cancer who have received chemotherapy rather than trastuzumab [**A(i)**] Positive nuclear PRPF38B expression was associated with reduced risk of relapse [HR (95% CI) = 0.64 (0.48–0.86); *p* = 0.002]. [**A(ii)**] Positive membranous PRPF38B expression was associated with increased risk of relapse [HR (95% CI) = 1.47 (1.07–2.01; *p* = 0.017]. Stratification of patients according to chemotherapy treatment revealed that [**B(i)**] ER-negative patients who showed positive nuclear PRPF38B expression were at lower risk of relapse compared to those negative for nuclear PRPF38B expression [HR (95% CI) = 0.49 (0.33–0.74); *p* = 0.0004]. [**B(ii)**] Patients with ER-negative tumours exhibiting positive membranous PRPF38B expression had a higher risk of relapse compared with patients with PRPF38B negative tumours [HR (95% CI) = 2.24 (1.40–3.57); *p* = 0.001]. Disease-free survival was assessed over a 5 year period. Relationships were determined using Kaplan-Meier univariate survival analysis.

Out of 693 cases of ER-negative breast cancer, 627 cases were suitable for the scoring of PRPF38B expression; 202 (32.2%) showed no staining, 132 (21.9%) demonstrated membranous expression, 285 (45.5%) showed nuclear expression and 27 (4.3%) showed cytoplasmic expression. Positive nuclear expression of PRPF38B was associated with reduced risk of relapse at the 5 year follow-up [HR (95% CI) = 0.64 (0.48–0.86); *p* = 0.002], whereas positive membranous PRPF38B expression was associated with increased risk of relapse at the 5-year follow-up [HR (95% CI) = 1.47 (1.07–2.01; *p* = 0.017] (Figure 3A). Stratification of patients according to chemotherapy treatment revealed that patients with ER-negative disease with positive nuclear PRPF38B expression who received cyclophosphamide, methotrexate, 5-fluorouracil (CMF) and anthracycline chemotherapy were at lower risk of relapse compared to those negative for nuclear PRPF38B expression [HR (95% CI) = 0.49 (0.33–0.74); *p* = 0.0004] (Figure 3B(i)). In contrast, patients with ER-negative tumours with positive membranous PRPF38B expression who received CMF and anthracycline chemotherapy had a higher risk of relapse compared with patients having membranous PRPF38B negative tumours [HR (95% CI) = 2.24 (1.40–3.57); *p* = 0.001] (Figure 3B(ii)).

### Relationship between cellular localization of PRPF38B expression and clinical outcome for patients with HER2-overexpression

**Figure 4:**
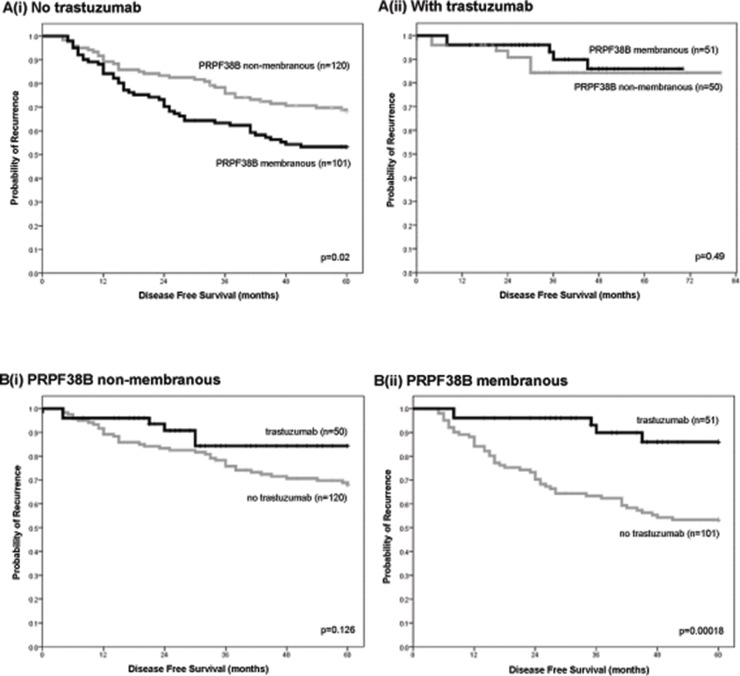
Relationships between PRPF38B expression and cellular localization and clinical outcome in patients with ER-negative/HER2-positive breast cancer in the presence or absence of trastuzumab treatment [**A(i)**] Positive membranous PRPF38B expression was associated with an increased risk of relapse in patients that were ER-negative and overexpressing HER2 who did not receive trastuzumab, as compared to patients with breast cancer that was negative for membranous PRPF38B expression [HR (95% CI) = 1.65 (1.07–2.53); *p* = 0.02]. [**A(ii)**] There was no significant difference in the survival of patients with ER-negative/HER2 overexpressing tumours that were treated with trastuzumab when stratified according to the localization of PRPF38B [HR (95% CI) = 1.65 (1.07–2.53); *p* = 0.49]. [**B(i)**] Non-membranous expression of PRPF38B was not associated with responsiveness to trastuzumab therapy in patients with ER-negative/HER2-positive breast cancer (*p* = 0.126). [**B(ii)**] Membranous PRPF38B expression is significantly associated with better disease-free survival (DFS) in patients with ER-negative/HER2-positive breast cancer who are treated with trastuzumab (*p* = 0.00018). Disease-free survival was assessed over a 5 year period. Relationships were determined using Kaplan-Meier univariate survival analysis.

For patients with HER2 over-expressing breast cancer who did not receive trastuzumab, the risk of relapse was greater for patients with membranous PRPF38B positive disease than those with membranous PRPF38B negative disease [HR (95% CI) = 1.65 (1.07–2.53); *p* = 0.02] (Figure 4A(i)). In patients with HER2 overexpressing breast cancer that received trastuzumab, there was no significant difference in 5 year disease-free survival (DFS) between those with positive and negative PRPF38B membranous expression [HR (95% CI) = 1.75 (1.7–2.5); *p* = 0.49] (Figure 4A(ii)). These findings infer that although negative membranous PRPF38B expression had no value in predicting the influence of trastuzumab therapy on disease-free survival, membranous PRPF38B expression did identify a cohort of patients with HER2 over-expressing disease that benefitted from trastuzumab therapy (5 year follow-up) [HR (95% CI) = 0.21 (0.08–0.52); *p* = 0.00018] (Figure 4(B)).

## DISCUSSION

We have used the SEREX approach to identify cancer testis antigens associated with human cancer by screening pooled human cancer sera against a human testis cDNA expression library. This approach has identified a novel splice variant of a previously characterised protein called PRPF38B (Supplementary Figure 1) which appears to elicit specific immune responses in the tumour-bearing host and which, at the RNA level, is significantly overexpressed in a significant number of breast cancer tissues when compared to their matched normal counterparts. In addition, isoforms of this protein have been detected in various breast cancer lines and its localization can be observed within the nuclear, cytoplasmic and membranous regions (Supplementary Figure 2). RT-PCR screening of a panel of normal tissues and tissues obtained from patients with different cancers suggest that levels of PRPF38B are low in the majority of essential organs and that elevated levels of this transcript (above the mean + 2 standard deviations) can be observed in 5/10 patients with kidney cancer, 2/2 patients with gastric cancer, 4/10 patients with prostate cancer and 5/10 individuals with benign prostatic hyperplasia (Supplementary Figure 3). PRPF38B is a unique component of the U4/U6.U5 tri small nuclear ribonuclear protein (snRNP) particle and is necessary for an essential step in late spliceosome maturation. Lee *et al.* (2003) reported on the isolation of a partial sequence of this gene (known as NY-SAR-27) following an immunomic analysis of human sarcoma. For this, sera from patients with sarcoma were used to screen a testis cDNA library and positive clones were identified and sequenced. To the best of our knowledge, no further work on this gene has been published [13]. Our subsequent detection of PRPF38B in a SEREX screen of sera from patients with breast cancer and the demonstration that it is over-expressed in breast cancer tissue led us to evaluate PRPF38B expression as a prognostic and therapeutic outcome predictor in a large series of breast cancers.

HER2 positivity is associated with early relapse after initial surgery for patients with breast cancer and a reduction in survival of up to 50%. Currently, anthracycline based chemotherapy in combination with trastuzumab is the standard of care for the treatment of patients with breast cancer overexpressing HER2. However, around 50% and 60% of these tumours are resistant to treatment with anthracycline chemotherapy and trastuzumab respectively. Furthermore, both drugs are associated with a small but significant risk of cardiotoxicity. The costs associated with these treatments places significant pressure on health service providers and a low-cost test which can identify those patients who will not respond to trastuzumab therapy would therefore provide significant benefits to patients, physicians and healthcare providers.

The clinically significant findings of this study are that: (1) Membranous PRPF38B expression is significantly associated with increased risk of relapse. (2) Membranous PRPF38B expression, lymph node stage, histological grade, tumour size, ER receptor positivity and Bcl2 status are powerful independent predictors for disease-free survival. (3) In the ER-negative patient cohort, nuclear localization of PRPF38B was associated with a reduced risk of relapse at 5 years, whereas those patients exhibiting membranous PRPF38B expression had an increased risk of relapse. (4) In patients with ER-negative/HER2-positive disease, membranous PRPF38B expression was associated with poor prognosis in patients that did not receive trastuzumab therapy, but was capable of identifying patients with ER-negative/HER2-positive disease that responded to trastuzumab therapy. Why membranous expression of PRPF38B is associated with an increased risk of relapse in patients with HER2+/ER-negative disease who did not receive trastuzumab, but is associated with a better disease-free survival in those patients treated with trastuzumab remains unknown. However, it could be that membranous PRPF38B expression is a surrogate biomarker for highly proliferative tumours.

Early studies have demonstrated that the relative concentrations of specific splicing factors can become altered during early pre-neoplasia and these become more pronounced during tumour formation [14]. A recent study has identified a set of pre-mRNA splicing factors that are required for sister chromatid cohesion in human cells and that loss of cohesion was an early cellular consequence of compromised splicing. Some cancers were shown to harbour mutations in these splicing factors [15].

The ability of PRPF38B expression to predict responsiveness to trastuzumab may be related to one of the postulated mechanisms of action of trastuzumab. However, it is also possible that membranous PRPF38B is simply a prognostic biomarker for responsiveness to trastuzumab therapy in the ER-negative/HER2-positive cohort of patients, rather than its presence having a direct influence on the actions of trastuzumab. Identifying the potential interactions between PRPF38B and HER2 is therefore important for future studies.

Although many (hundreds) of putative biomarkers have been proposed to date, these studies lack evidence of applicability to a clinical question. To date, the most widely investigated tumour tissue biomarkers are UPA/PAI-1 [16], ki67 [17], the serum biomarkers CA 15-3 and carcinoembryonic antigen [18] and gene expression profiles such as Oncotype DX [16, 19]. Other established biomarkers include p53 [20], HER2/neu [21] and BRCA1/BRCA2 [22]. We have previously published on the identification of multiple breast cancer biomarkers [23, 24] and work on these includes their utility as potential vaccine candidates [25–27] and as a prognostic biomarker and chemotherapy sensitivity predictor in breast cancer [28]. Although the plethora of breast cancer biomarkers is steadily increasing, we believe we are the first to report on a biomarker that can aid clinicians in their decision-making process regarding the administration of trastuzumab therapy to ER-negative/Her2-positive breast cancer patients.

The identification of PRPF38B as a potential biomarker for predicting patient response to trastuzumab therapy aligns this biomarker well for use alongside other commercially available tests. Although Oncotype DX is one such available platform, this is a genomic-based approach and is not utilised in ER-negative patients, nor as a means for predicting a patient's response to trastuzumab. An immunohistochemistry based approach, performed on tumour material that is resected at the point of surgical intervention, could be undertaken at the same time as staining for other clinically-relevant markers (i.e. Her2/neu status). If PRPF38B was included in this biomarker panel then the results would aid the clinical decision-making process with regards to potentially avoiding the administration of costly trastuzumab that would be of no clinical benefit to the patient, but which may cause significant unwanted side-effects.

In terms of the potential economic costs and benefits associated with the addition of this biomarker test into clinical practice, it is known that about 60 patients per million population per year present with HER2-positive breast cancer and these are routinely treated with trastuzumab (some indication as to the prevalence of PRPF38B alone and in combination with ERBB2 (Her2) is given in Supplementary Table 2). However, of these patients, less than 50% will respond and this represents a significant over-treatment of patients, costing the NHS £73 million (US$124 million) for trastuzumab alone. In terms of patient benefit, the use of this proposed biomarker as a predictive test will avoid a large number of patients undergoing unnecessary treatments which are disruptive to their lives and may result in unnecessary toxicity, with adverse consequences on their quality of life. In essence, the identification of this model has the potential to underpin a new, precision medicine-based approach for treating this form of breast cancer which will enable these patients to be treated with more effective strategies, thereby improving outcomes and quality of life.

In the wider context, the significance of membranous PRPF38B as a biomarker for response to trastuzumab therapy may not be solely restricted to breast cancer. HER2 overexpression has been described in a variety of tumours including, but not limited to, bladder, breast, cervix, colorectal, endometrium, oesophagus, gastric, head and neck, liver, melanoma, lung (NSCLC), osteosarcoma, ovary, prostate and salivary duct [8]. Trastuzumab is approved for the treatment of breast, gastric and gastroesophageal junction cancer by the US Federal Drugs Administration (FDA), and there are mounting anecdotal reports of responses to HER2-targeted agents in patients with NSCLC [29–33] and cancer of the salivary duct [34–38].

Finally, the membranous expression of PRPF38B may have a potential utility as an immunotherapeutic target. Numerous studies have emphasised the variable clinical benefits of HER2-targeted therapies [21, 39, 40], and there is a significant emphasis on developing new immunomodulatory drugs and HER2-targeted therapies that can be used to augment responses and clinical outcome in combination [39]. The identification of membranous PRPF38B expression in breast cancer affords the opportunity to develop an antibody targeting strategy for therapeutic intervention.

## CONCLUSIONS

This study demonstrates that the expression of PRPF38B is an independent prognostic marker in breast cancer. Nuclear PRPF38B expression was significantly associated with a decreased risk of relapse at 5 years in patients with ER-negative disease and patients with ER-negative disease that had received chemotherapy, rather than trastuzumab. Furthermore, membranous expression of PRPF38B was significantly associated with a poor prognosis at the 5 year follow-up in patients with ER-negative/HER2-positive breast cancer who were treated prior to 2006 and did not receive trastuzumab therapy. After 2006, patients with ER-negative/HER2-positive breast cancer were treated with trastuzumab and, in these patients, membranous expression of PRPF38B in the primary cancer was significantly associated with better disease-free survival (DFS) at the 5 year follow-up. This study therefore reveals and highlights the potential importance of membranous PRPF38B expression as a parameter for predicting therapeutic response to trastuzumab therapy in patients with ER-negative/HER2-positive breast cancer.

These findings suggest that further validation in larger, independent cohorts of patients with ER negative/HER2-positive breast cancer is warranted. These will need to focus on evaluating the value of membranous PRPF38B expression as a tool for stratifying patients for trastuzumab therapy rather than other HER2 targeting strategies. Exploring the utility of this biomarker as an indicator of response to trastuzumab therapy in other cancer types, for which trastuzumab is currently approved as a therapeutic option, is also indicated.

## PATIENTS AND METHODS

### Expression of PRPF38B in early stage primary invasive breast cancer and normal breast tissue

The expression of PRPF38B in tissues from a consecutive series of 1650 patients with primary invasive breast carcinomas who were diagnosed between 1986 and 1999, and entered into the Nottingham Tenovus Primary Breast Carcinoma series was determined using immunohistochemistry; 1388 cores within the TMAs were suitable for immunohistochemical (IHC) staining and assessment. Patients were randomised into two equal cohorts using a double random number sort, for which alternate cases were allocated into a training set and a test set. Both cohorts contained a similar proportion of patients with regards to clinicopathological features, treatment and survival data (Supplementary Tables 1 and 3).

Prior to 1989, patients received standard surgery (mastectomy or wide local excision) with radiotherapy, after which patients received adjuvant therapy on the basis of prognostic and predictive parameters, as indicated using the Nottingham Prognostic Index (NPI), ERα and menopausal status. However, patients within the ‘good’ NPI group (≤3.4) did not receive adjuvant therapy. Hormone therapy (HT) was prescribed to post-menopausal patients with ERα positive and NPI scores >3.4 (moderate and poor prognostic group), whereas ERα negative patients received classical cyclophosphamide, methotrexate, 5-flouracil (CMF) chemotherapy. Pre-menopausal patients within the moderate and poor prognostic groups were candidates for CMF chemotherapy, and patients with ERα positive tumours were also offered hormone therapy. Median follow-up was 111 months (range 1 to 233 months).

PRPF38B protein expression was also evaluated in 40 normal breast tissue biopsy specimens removed from women (age range 25-60 years) with no family history of breast cancer that underwent reduction mammoplasty, primarily due to cosmetic reasons.

### PRPF38B expression in ER-negative breast cancer

To evaluate the relationship between PRPF38B expression and clinical outcome after receiving adjuvant chemotherapy, we analysed its expression in a consecutive series of 693 patients with early stage ER-negative breast cancer who had been diagnosed and managed at Nottingham City Hospital between 1999 and 2007. This series included 303 patients treated before 2000 who were either chemotherapy-naïve or received adjuvant CMF, and 390 patients who were treated after 2000 and were either chemotherapy-naïve or were treated with anthracycline-based adjuvant chemotherapy (anthracycline-ACT). Disease-free survival was used as a primary endpoint.

### PRPF38B expression in HER2-overexpressing breast cancer

To evaluate the relationship between PRPF38B expression and clinical outcome in HER2-positive breast cancer, we analysed its expression in a consecutive series of 443 patients with early stage breast cancer over-expressing HER2 who had been diagnosed and managed at Nottingham City Hospital between 1986 and 2010. This series included 303 patients who were chemotherapy-naïve or received adjuvant CMF before 2006, and 140 patients who were treated after 2006 and received trastuzumab therapy. Disease-free survival was used as a primary endpoint.

The Reporting Recommendations for Tumor Marker Prognostic Studies (REMARK) criteria were adhered to throughout this study. All work was approved by the Nottingham Research Ethics Committee (approval number C1080301).

### Tissue microarrays (TMAs) and immunohistochemistry (IHC)

Breast tumour TMAs were constructed using 6 replicate 0.6 mm cores from the centre and periphery of the tumours of each patient. The TMAs were immunohistochemically profiled for the expression of PRPF38B and other clinically significant markers, as described previously [41]. The primary antibodies, clone, source, optimal dilution and scoring system used are summarised in Supplementary Table 4. TMAs were subjected to microwave antigen retrieval in 0.01 M pH 6.0 citrate buffer prior to an overnight incubation at room temperature with a monospecific rabbit antibody to PRPF38B (1:1000) which was raised against the 17 amino acid sequence IEQESQEKQHKNKDETV conjugated to KLH (custom prepared by Pacific Immunology, USA). Two rabbits received 4 immunisations, and 4 production bleeds from each animal were pooled and antibody affinity purified using custom affinity columns against the synthesised peptide. The specificity of the monospecific PRPF38B antibody was confirmed by demonstrating that specific PRPF38B IHC staining and the presence of bands in immunoblots were completely abrogated by pre-incubating the antibody with the relevant peptide, but not with an irrelevant peptide (data not shown). Immunohistochemical staining was performed using a Novolink detection kit according to the manufacturer's protocol (Leica Microsystems, UK) on a DakoCytomation Techmate 500 Plus (DakoCytomation,UK) automatic immunostainer.

The expression of HER2, oestrogen and progesterone receptors was assessed according to the most recent American Society of Clinical Oncology/College of American Pathologists (ASCO/CAP) guidelines [42]. HER2 status of the tissues that were used in the current study was assessed using immunohistochemistry and fluorescence *in situ* hybridisation (FISH), as previously described [43]. Positive and negative (omission of the primary antibody and IgG-matched serum) controls were included in each immunohistochemistry run.

### Evaluation of PRPF38B staining

Tumour cores were evaluated by two pathologists who were blinded to the clinicopathological characteristics of the patients (co-authors: TAF, IOE). Intra- and inter-observer agreements were excellent (*k* > 0.8; Cohen's *k* and multirater *k*-tests, respectively). Whole field inspection of the core was scored and intensities of nuclear, cytoplasmic and membranous staining were grouped as follows: 0 = no staining, 1 = weak staining, 2 = moderate staining and 3 = strong staining. The percentage of each category was estimated.

### Survival data and statistics

Data on overall survival, disease-free survival, and development of loco-regional and distant metastases (DM) were maintained on a prospective basis. DFS was defined as the number of months from diagnosis to the occurrence of local recurrence, local lymph node (LN) relapse or DM relapse. Breast cancer specific survival (BCSS) was defined as the number of months from diagnosis to the occurrence of breast cancer-related death. Survival was censored if the patient was still alive at the time of analysis, lost to follow-up, or died from other causes.

Pearson's Chisquare, Fisher's exact, Student's *t*-test and ANOVA oneway tests were performed using SPSS 17.0 statistical software (SPSS Inc, USA). Cumulative survival probabilities were estimated using the Kaplan-Meier method and a log rank test was used to assess significant differences between survival rates. Multivariate analysis for survival was performed using a Cox proportional hazard regression model. Each variable was assessed in univariate analysis as a continuous and categorical variable, and the two models were compared using an appropriate likelihood ratio test. Hazard ratios (HR) and 95% confidence intervals (CI) were estimated for each variable. All tests were two-sided with a 95% CI and a *p* value < 0.05 was considered to reflect statistical significance. For multiple comparisons, *p* values were adjusted according to Holm-Bonferroni method.

## SUPPLEMENTARY MATERIALS FIGURES AND TABLES



## Abbreviations

IDCNST: invasive ductal carcinoma of no specific type
ER: oestrogen receptor
BC: breast cancer
PR: progesterone receptor
ORR: overall response rates
SEREX: serological analysis of recombinant cDNA expression libraries
DFS: disease-free survival
HR: hazard ratio
CI: confidence interval
CMF: cyclophosphamide methotrexate 5-fluorouracil
ADCC: antibody dependent cellular cytotoxicity
NSCLC: non small cell lung carcinoma
TMA: tumour microarray
NPI: Nottingham prognostic index
IHC: immunohistochemistry
DM: distant metastases
LN: lymph node
BCSS: breast cancer specific survival
