# Supplementary material for: The localization of pre mRNA splicing factor PRPF38B is a novel prognostic biomarker that may predict survival benefit of trastuzumab in patients with breast cancer overexpressing HER2
Source: Oncotarget. 2017 Nov 18;8(68):112245–57. doi: 10.18632/oncotarget.22496 (PMC5762507; doi:10.18632/oncotarget.22496)
Supplement: Supplementary file 1 [file oncotarget-08-112245-s001.pdf]

The localisation of pre mRNA splicing factor PRPF38B is a novel prognostic biomarker that may predict survival benefit of trastuzumab in patients with breast cancer overexpressing HER2

SUPPLEMENTARY MATERIALS

A

(i)

TTTCCAATTATTTGTTCATTTATTTATTTTCTACATAACTAAATTAGAAACCTCACTGCTTCATGGCAGTTGGTTTGCT
ATTGCTTCCAGTTTTATTAGGGCTTCATTTTATATTAGAGCTGTTAAAAGATAACCTTTAGACAGGAATTATCTAAAGT
AGATCATATGTAGCTAGGTTATGGTGCAAGGTGTATGATGTGTGCAAATATGTCCACAGAAATAAATACATAGTAGG
TATGTGGAATGTAAATTTAAGTCAATCGTTCCGCATAGTTTAGAAATGTAAGGGGCTTTTTCATATTGTTAACTGAGT
GAGATCAGTTCCCTTTATGCCTGTGAGGCTGCAGGGTTTGTTCTCACTTGCATGCACACACTAAGCCCAAATATTTC
TGTTCATTCATTGTCAGATCAGGATATGAAAATAAAATTTTTCTGTTAGTTTTTTTTGTATTGAGATTCCAAAGATGGT
AATATTTTTATAATATTCATGTATATATGGAAATACTTTTTTTGACGGCTAGGGTATCTTTTGTGTTTCTGTAGGACCT
AGATGTGAAGGCTGGTGGAGGCTGTGTAATGACCATTGGAGAAATGCTACGATCTTTTCTCACAAAACTGGAGTGG
TTTTCTACCTTGTTTCCAAGAATTCCAGTTCCAGTTCAAAAGAATATTGATCAACAGATTAAAACCCGACCTAGAAAA
ATCAAGAAAGATGGGAAGGAAGGTGCTGAGGAAATAGACAGACATGTTGAACGCAGACGTTCAAGGTCTCCAAGG
AGATCTCTGAGTCCACGGAGGTCCCCAAGAAGGTCAAGAAGTAGAAGTCATCATCGGGAGGGCCATGGGTCTTCT
AGTTTTGACAGAGAATTAGAAAGAGAGAAAGAACGCCAGCGACTAGAGCGTGAAGCCAAAGAAAGGGAGAAAGAA
CGGCGAAGATCCCGAAGTATTGACCGGGGGTTAGAACGCAGGCGCAGCAGAAGTAGGGAAAGGCATAGAAGTCG
CAGTCGAAGTCGTGATAGGAAAGGGGATAGAAGGGACAGGGATCGAGAAAGAGAGAAAGAAAATGAGAGAGGTA
GAAGACGAGATCGTGACTATGATAAGGAAAGAGGAAATGAACGAGAAAAAGAGAGAGAGCGATCAAGAGAAAGGT
CCAAGGAACAGAGAAGTAGGGGAGAGGTAGAAGAGAAGAAACATAAAGAAGACAAAGATGATAGGCGGCACAGA
GATGACAAAAGAGATTCCAAGAAAGAGAAAAAACACAGTAGAAGCAGAAGCAGAGAAAGGAAACACAGAAGTAGG
AGTCGAAGTAGAAATGCAGGGAAACGAAGTAGAAGTAGAAGCAAAGAGAAATCAAGTAAACATAAAAATGAAAGTA
AAGAAAAATCAAATAAACGAAGTCGAAGTGGCAGTCAAGGAAGAACTGACAGTGTTGAAAAATCAAAAAAACGGGA
ACATAGTCCCAGCAAAGAAAAATCTAGAAAGCGTAGTAGAAGCAAAGAACGTTCCCACAAACGAGATCACAGTGAT
AGTAAGGACCAGTCAGACAAACATGATCGTCGAAGGAGCCAAAGTATAGAACAAGAGAGCCAAGAAAAACAGCAT
AAAAACAAAGATGAGACTGTGTGAAAATATTTTGTAAAAGTGGATCACATTGAATCCTATAAATGATTAAATCTGCTT
TTTTCCCCCACGTTGAGATTGTGCAGTAGTTCGCACTCCTCAAGCTCTCCCTGTAGGCTGCATTTTCATTTCCTCTT
TCGTGTAGGGAAGTGCCTTTGTAATTCCATTTATTGCATTGGTGTTTTCACCCAATTGTTAAGTTTGATACATGATGC
ACAGATTGTTCTTGCATTTTTATTGTTTGTTTTTGAAATGTACAGTCTGTACATATGTCCTGAAAATGTTTTAATTCCT
TTGGCATGGTTGCCATGTTGGTTAAATTTGTATAAGGCAATAAACTGCCACTAATCCAAAAAAAAAAAAAAAAAAAAA
AAAAA

(ii)

MTIGEMLRSFLTKLEWFSTLFPRIPVPVQKNIDQQIKTRPRKIKKDGKEGAEEIDRHVERRRSRSPRRSL
SPRRSPRRSRSRSHHREGHGSSSFDRELEREKERQRLEREAKEREKERRRSRSIDRGLERRRSRSR
ERHRSRSRSRDRKGDRRDRDREREKENERGRRRDRDYDKERGNEREKERERSRERSKEQRSRGEV
EEKKHKEDKDDRRHRDDKRDSKKEKKHSRSRSRERKHRSRSRSRNAGKRSRSRSKEKSSKHKNESK

(iii)

Accession	Description	Query coverage
BAA91546.1	unnamed protein product [*Homo sapiens*]	100%
NP_060531.2	pre-mRNA splicing factor 38B [*Homo sapiens*]	100%
CAH72070.1	PRP38 pre-mRNA processing factor 38 (yeast) domain containing B [*Homo sapiens*]	100%

B

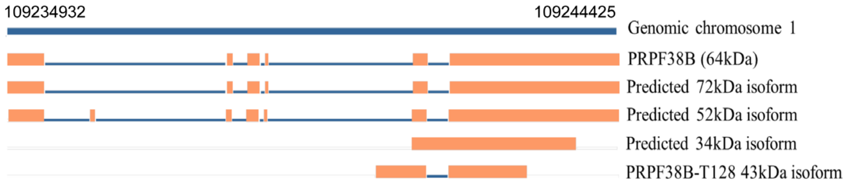

Supplementary Figure 1: Genomic characterisation of PRPF38BT128. (**A**) (i) The nucleotide sequence of PRPF38B-T128 (ii) The putative protein sequence of PRPF38B-T128. (iii) Blast alignment of the PRPF38B-T128 nucleotide sequence against the *Homo sapiens* database on the NCBI website, 100% sequence homology was identified to the PRPF38B gene [GenBank: NP_060531.2]. (**B**) The nucleotide sequence of PRPF38B, its related predicted isoforms (that could potential cross-react with the antibody used) [AceView: aAug10 (72 kDa), bAug10 (52 kDa) and aAug10-unspliced (34 kDa) and PRPF38B-T128 were aligned against the genomic sequence from *Homo sapiens* chromosome 1, GRCh37.p9 Primary Assembly (GenBank: gi_224589800) using the Spidey alignment tool.

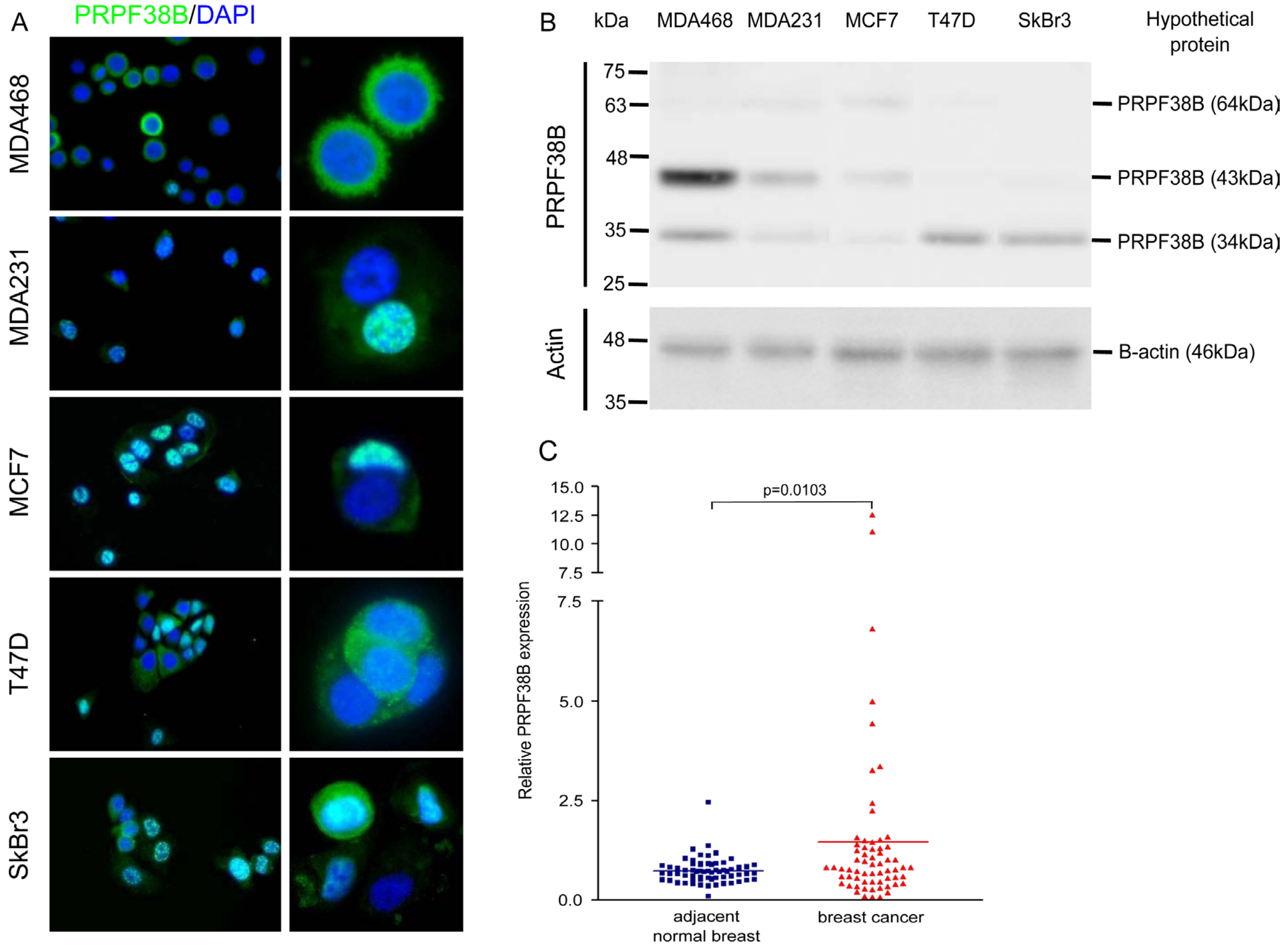

Supplementary Figure 2: (**A**) Immunofluorescent staining of 5 breast cancer cell lines (see Supplementary Table 4 for cell phenotype) using a monospecific antibody to PRPF38B (green). Cell nuclei are stained with DAPI (blue). PRPF38B protein was expressed in a variety of subcellular locations in all the cell lines tested. Magnification 20× and 100× respectively. (**B**) Immunoblotting of the 5 breast cancer cell lines using antibody against PRPF38B. β-actin was measured as a loading control. Immunoblotting (IB) reveals 3 distinct bands at 64kDa, 43kDa and 34kDa corresponding to the full length of PRPF38B, the putative 43kDa PRPF38BT128 isoform and the predicted PRPF38B 34kDa isoform respectively. These immunoblots are representative of three independent experiments. (**C**) Relative expression of PRPF38B mRNA in paired breast cancer and adjacent normal tissues (n = 63). The quantification of PRPF38B mRNA expression was performed by qPCR. Each PCR run was performed in triplicate and replicates for each sample were included in each run. HPRT1 and TBP were used as housekeeping genes for normalising the ct values.

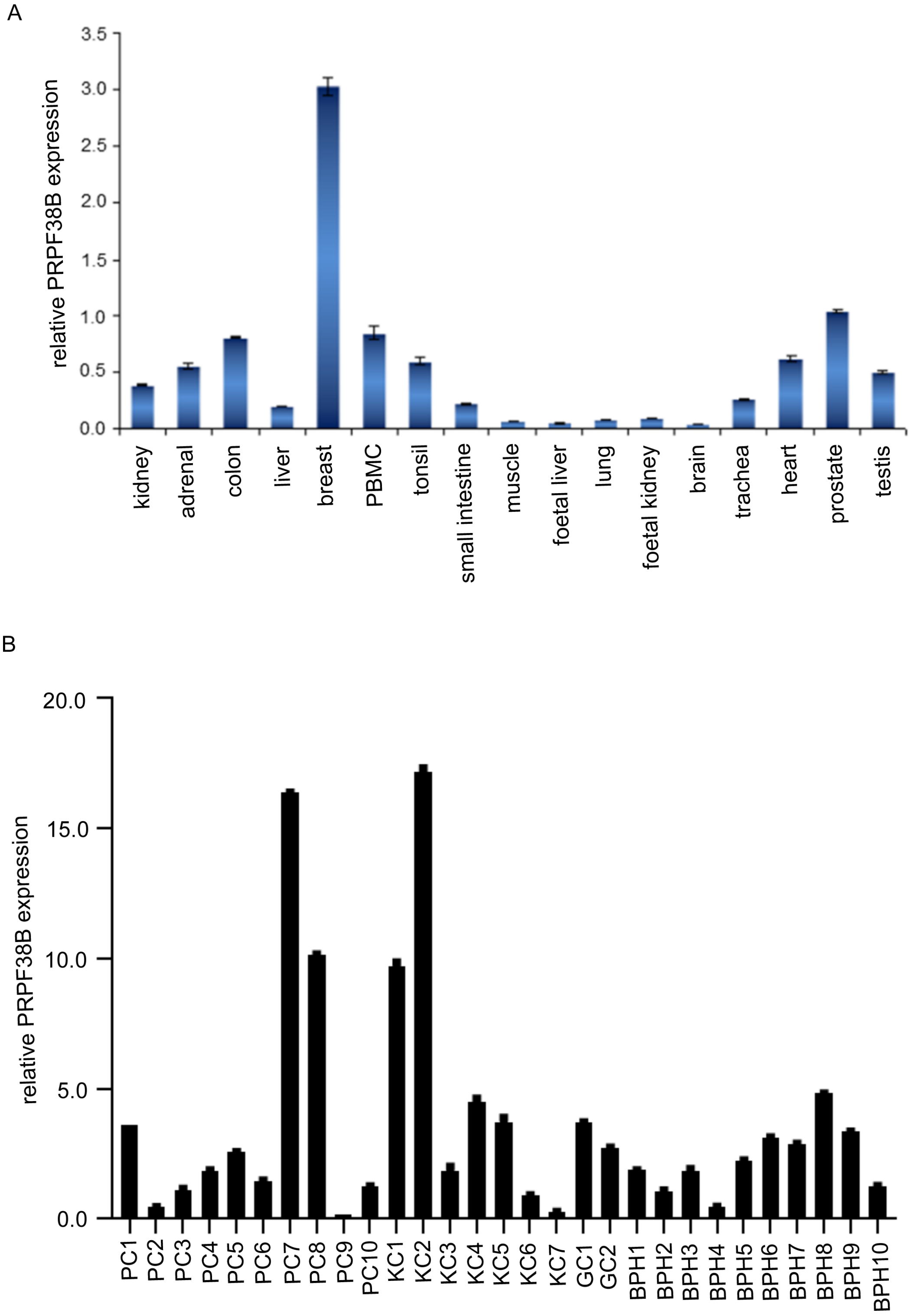

Supplementary Figure 3: Relative expression of PRPF38B mRNA in a panel of (**A**) normal tissues (purchased from Clontech) and (**B**) malignant and diseased tissues (PC = prostate cancer, KC = kidney cancer, GC = gastric cancer and BPH = benign prostatic hyperplasia. The quantification of PRPF38B mRNA expression was performed by qPCR. Each PCR run was performed in triplicate and replicates for each sample were included in each run. HPRT1 and TBP were used as housekeeping genes for normalising the ct values.

Supplementary Table 1: Disease parameters and treatment regimen

Variable		Training Set *n* (%)	Test Set *n* (%)	*p* value
PRPF38B membranous expression	Negative	613 (89.1)	616 (89.5)	0.793
	Positive	75 (10.9)	72 (10.5)	
Tumour size	T1 a+ b (≤1.0)	64 (9.4)	84 (12.2)	0.207
	T1 c (>1.0–2.0)	356 (52.5)	330 (48.1)	
	T2 (>2.0–5.0)	244 (36.0)	253 (36.9)	
	T3 (>5)	14 (2.1)	19 (2.8)	
Lymph node stage	Negative	420 (61.6)	410 (59.8)	0.776
	Positive (1–3 nodes)	203 (29.8)	212 (30.9)	
	Positive (>3 nodes)	59 (8.7)	64 (9.3)	
Tumour grade	Low grade (G1)	114 (16.8)	117 (17.1)	0.964
	Intermediate grade (G2)	231 (34.1)	229 (33.4)	
	High grade (G3)	333 (49.1)	340 (49.6)	
Lymphovascular invasion	No	456 (67.3)	447 (65.6)	0.528
	Yes	222 (32.7)	234 (34.4)	
Tumour type	IDC-NST	358 (59.4)	350 (58.8)	0.916
	Medullary	122 (20.2)	128 (21.5)	
	Tubular	14 (2.3)	14 (2.4)	
	Lobular	64 (10.6)	55 (9.2)	
	Others	45 (7.5)	48 (8.1)	
Mitotic index	M1 (low; mitoses <10)	252 (37.3)	239 (35.0)	0.413
	M2 (medium; mitoses 10–18)	130 (19.3)	123 (18.0)	
	M3 (high; mitoses >18)	293 (43.4)	321 (47.0)	
Bilateral phenotype	Unilateral	580 (95.9)	569 (94.8)	0.394
	Bilateral	25 (4.1)	31 (5.2)	
Oestrogen receptor	Negative	178 (26.4)	180 (26.7)	0.915
	Positive	496 (73.6)	495 (73.3)	
Progesterone receptor	Negative	217 (42.5)	261 (40.7)	0.493
	Positive	366 (57.5)	381 (59.3)	
HER2 receptor	Negative	587 (86.8)	605 (89.6)	0.111
	Overexpression	89 (13.2)	70 (10.4)	
Basal-like	No	585 (88.5)	575 (87.5)	0.583
	Yes	76 (11.5)	82 (12.5)	
Triple negative	No	553 (82.7)	539 (80.7)	0.351
	Yes	116 (17.3)	129 (19.3)	
**Treatments:**				
Operation type	WLE lumpectomy	281 (40.8)	299 (43.5)	0.280
	Mastectomy	404 (58.7)	381 (55.4)	
	Unknown	3 (0.4)	8 (1.1)	
Chemotherapy	No chemotherapy	563 (84.5)	579 (86.4)	0.352
	Received CMF	103 (15.5)	90 (13.4)	
	Anthracycline	0 (0)	1 (0.1)	
Endocrine therapy	No	397 (60.3)	406 (61.1)	0.890
	Tamoxifen	230 (35.0)	229 (34.4)	
	Tamoxifen + Zoladex	24 (3.6)	21 (3.2)	
	Others	7 (1.1)	9 (1.4)	

There is no significant difference (p value > 0.05) between disease parameters and treatment regimens following randomisation into two equal cohorts using a double random number sort. No significant difference in PRPF38B membranous expression, tumour size, lymph node stage, tumour grade, lymphovascular invasion, tumour type, mitotic index, bolateral phenotype, oestrogen receptor, progesterone receptor, HER2 receptor, basal-like, triple negative, operation type, chemotherapy and endocrine therapy between the training and test data sets was observed, as determined using Pearson's Chi-squared tests.

Supplementary Table 2: Prevalence of PRPF38B and ERBB2 in METABRIC [a] and TCGA [b] datasets

Gene	PRPF38B		PRPF38B + ERBB2	
**Dataset**	**METABRIC**	**TCGA**	**METABRIC**	**TCGA**
Prevalence	124/2509 (5%) cases	35/526 (7%) cases	PRPF38B (5%)<br>ERBB2 (14%)<br>449/2509 cases	PRPF38B (7%)<br>ERBB2 (17%)<br>114/526 cases

The prevalence of PRPF38B on its own and in combination with ERBB2 (Her2) in the METABRIC and TCGA datasets is given. Using the METABRIC dataset (2509 tumour samples in total), PRPF38B mRNA expression was upregulated in 47 patients and downregulated in 77 patients. When PRPF38B and ERBB2 were investigated together the following prevalence was observed: 5 patients had mRNA upregulation of both PRPF38B and ERBB2 mRNA. 38 patients had upregulation of PRPF38B mRNA and no change in ERBB2 mRNA expression. 67 patients had downregulation of PRPF38B mRNA and no change in ERBB2 mRNA expression. 16 patients had upregulation of PRPF38B mRNA and downregulation in ERBB2 mRNA expression. 4 patients had downregulation of PRPF38B mRNA and upregulation in ERBB2 mRNA expression. Using the TCGA dataset (526 tumours with mRNA data–Agilent microarray), PRPF38B mRNA expression was upregulated in 15 patients and downregulated in 20 patients. When PRPF38B and ERBB2 were investigated together the following prevalence was observed: 11 patients had upregulation of PRPF38B mRNA and no change in ERBB2 mRNA expression. 15 patients had downregulation of PRPF38B mRNA and no change in ERBB2 mRNA expression. 4 patients had upregulation of PRPF38B mRNA and downregulation in ERBB2 mRNA expression. 5 patients had downregulation of PRPF38B mRNA and upregulation of ERBB2 mRNA expression. **a.** Pereira B, Chin SF, Rueda OM, Vollan HKM, Provenzano E, Bardwell HA, Pugh M, Jones L, Russell R, Sammut SJ, Tsui DWY, Liu B, Dawson SJ, *et al.* The somatic mutation profiles of 2,433 breast cancers refines their genomic and transcriptomic landscapes. Nat Commun. 2016; 7:11479. https://doi.org/10.1038/ncomms11479. **b.** Ciriello G, Gatza ML, Beck AH, Wilkerson MD, Rhie SK, Pastore A, Zhang H, McLellan M, Yau C, Kandoth C, Bowlby R, Shen H, Hayat S, *et al*. Comprehensive Molecular Portraits of Invasive Lobular Breast Cancer. Cell. 2015; 163:506–19. https://doi.org/10.1016/j. cell.2015.09.033.

Supplementary Table 3: Patient demographics

**Clinical features:**		**Training Set** ***n* (%)**	**Test Set** ***n* (%)**	***p* value**
**Age**	≤50 years	217 (35.9)	226 (37.7)	0.503
	>50 years	388 (64.1)	373 (62.3)	
**Family history**	No	450 (78.3)	430 (76.4)	0.448
	Yes	125 (21.7)	133 (23.6)	
**Survival follow up**				
	Mean (months)	121	121	NS
	Median (months)	130	124	
	Range (months)	5–239	0–243	
**Survivals**	Alive and well or loss follow	494 (71.8)	493 (71.7)	0.952
	Dead from disease	194 (28.2)	195 (28.3)	
**Recurrence**	Yes	403 (58.6)	396 (57.6)	0.702
	No	285 (41.4)	292 (42.4)	
**Distant metastases**	Yes	473 (68.8)	459 (66.7)	0.419
	No	215 (31.3)	229 (33.3)	
**Prognosis on the basis of NPI**				
Excellent	(2.08–2.40)	71 (10.5)	83 (12.1)	0.506
Good	(2.42–3.40)	144 (21.2)	120 (17.5)	
Moderate I	(3.42–4.40)	193 (28.5)	196 (28.6)	
Moderate II	(4.42–5.40)	160 (23.6)	168 (24.5)	
Poor	(5.42–6.49)	89 (13.1)	90 (13.1)	
Very poor	(≥6.50)	21 (3.1)	28 (4.1)	
Unknown				

There is no significant difference (*p* value < 0.05) between various patient demographics following randomisation into two equal cohorts using a double random number sort. No significant difference was observed in age, family history, survival follow up, survivals, recurrence, distant metastases and prognosis on the basis of the Nottingham Prognostic Index (NPI) between the training and test data sets, as determined using Pearson's Chi-squared tests.

Supplementary Table 4: Antigens, primary antibodies, clone, source, optimal dilution and scoring system used for each immunohistochemical marker

Antigen	Antibody	Clone	Source	Antigen Retrieval	Dilution / Incubation Time	Distribution	Scoring system	Cut-offs
p53	Mouse MAb anti p53	DO7	Novocastra	Citrate pH6	1:50<br>60 min	Nuclear	% of positive cells	≤20% (negative)<br>>20% (High)
Bcl2	Mouse MAb anti-Bcl2	124	Dako-Cytomation	Citrate pH6	1:100<br>60 min	Cytoplasm	% of positive cells	>10% (positive
BRCA1	BRCA1	MS110	Calbiochem	Citrate pH6	1:100<br>60 min	Nuclear	% of positive cells	<25% (negative)
ATM	Rabbit MAb anti-ATM	Y170	Abcam	Citrate pH6	1:100<br>18 hours	Nuclear	% of positive cells	<25% (negative)
p27	anti-p27	SX53G8	Dako-Cytomation	Citrate pH6	1:50<br>60 min	Nuclear	% of positive cells	≥10% (positive)
Vimentin	Mouse MAb anti-vimentin	Vim 3B4	Dako-Cytomation	Citrate pH6	1:250<br>60 min	Cytoplasm	% of positive cells	≥10% (positive)
Bax	Rabbit anti-Bax	Polyclonal	Abcam	Citrate pH6	1:1000<br>60 min	Cytoplasm	% positive cells	≥10% (positive)
ER	Mouse MAb anti-ER-α	SP1	Dako-Cytomation	Citrate pH6	1:150<br>30 min	Nuclear	Allred score	≥3 (positive)
ER	Mouse MAb anti-ER-α	EP1	Dako-Cytomation	Citrate pH6	1:80<br>30 min	Nuclear	% positive cells	≥1% positive
PR	Mouse MAb anti-PR	PgR636	Dako-Cytomation	Citrate pH6	1:125<br>30 min	Nuclear	% positive cells	≥1% positive
EGFR	Mouse MAb anti-EGFR	31G7	Zymed Laboratories	Proteinase K, 370C for 8 min	1:30<br>60 min	Membrane	0-3 as HER2	0 or +1 (negative)<br>+2 or +3 (positive)
CK14	Mouse MAb anti-Ck14	LL002	Novocastra	Citrate pH6	1:40<br>60 min	Cytoplasm	% of positive cells	≥10% (positive)
Ck5/6	Mouse MAb anti-Ck5/6	D5/161B4	Dako-Cytomation	EDTA pH8	1:100<br>60 min	Cytoplasm	% of positive cells	≥10% (positive)
Ck17	Mouse MAb anti-Ck17	E3	Dako-Cytomation	Citrate pH6	1:100<br>60 min	Cytoplasm	% of positive cells	≥10% (positive)
Ck18	Mouse MAb anti-Ck18	DC10	Dako-Cytomation	Citrate pH6	1:100<br>60 min	Cytoplasm	% of positive cells	≥10% (positive)
HER2	Rabbit antihuman c-erbB2	polyclonal	Dako-Cytomation	None	1:400<br>60 min	Membrane	See text	See text
Ki67	Mouse MAb anti-Ki-67	MIB1	Dako-Cytomation	Citrate pH6	1:300<br>60 min	Nuclear	% of positive cells	0–30% (low)<br>>30% (high)
TOP2A	Mouse MAb TOP2A	KiS1	Dako-Cytomation	Citrate pH6	1:100<br>60 min	Nuclear	% of positive cells	>25% (positive)
p21	Mouse MAb anti-p21	SW118	Dako-Cytomation	Citrate pH6	1:50<br>60 min	Nuclear	% of positive cells	≥10% (positive)
MDM2	Mouse MAb anti-MDM2	1B10	Novocastra	Citrate pH6	1:200<br>60 min	Nuclear	% of positive cells	≥10% (High)
MDM4	Affinity purified rabbit anti-HdmX/MDM4	IHC-00108	Bethyl Labs	Citrate pH6	1:100<br>60 min	Nuclear	% of positive cells	0–20% (Low)<br>>20% (High)
HER3	Mouse MAb anti-HER3	RTJ1	Novocastra	Citrate pH6	1:20<br>60 min	Cytoplasm and Membrane	H score	H score <150
HER4	Rabbit antihuman c-erbB4	polyclonal	Neo Marker	None	6:4<br>60 min	Cytoplasm	H score	H score <100
P-cadherin	Mouse MAb anti-P-cadherin	Clone 56	BD Bioscience	None	1:200<br>60 min	cytoplasm	% of positive cells	>5% positive
E-cadherin	Mouse MAb anti-E-cadherin	HERCD-1	Zymed Laboratories	Citrate pH6	1:100<br>60 min	Membrane	H score	H score ≤100
SPAG5	Rabbit anti-SPAG5	polyclonal	Sigma-Aldrich	Citrate pH6	1:50<br>60 min	Cytoplasmic	% of positive cells	>10% (positive)

All sections were pre-treated with microwave antigen retrieval using 0.1% (v/v) citrate buffer (pH 6), except for HER2 (no pre-treatment) and EGFR (pre-treated with protease for 10 min).

MAb: Monoclonal antibody; MDM2: murine double minute 2; MDM4: murine double minute 4; ATM: ataxia telangiectasia mutated; BRCA1: BC 1, ER: oestrogen receptor; PR: progesterone receptor; CK: cytokeratin; EGFR: epidermal growth factor; TOP2A: Topoisomerase II alpha, HER2 (ERBB2): v-erb-b2 erythroblastic leukaemia viral oncogene homolog 2, neuro/glioblastoma derived oncogene homolog (avian), HER3 (ERBB3): v-erb-b2 erythroblastic leukaemia viral oncogene homolog 3 (avian), HER4 (ERBB4): human epidermal receptor 4, Bcl2: B-cell CLL/lymphoma 2, Bax: BCL2-associated X protein , SPAG5: sperm associated antigen 5, KIF2C: kinesin family member 2C.
